# Risk factors associated with the use of red blood cells in elective cardiac surgeries: A patient blood management (PBM) view

**DOI:** 10.1016/j.htct.2025.103987

**Published:** 2025-09-17

**Authors:** Tainá Miotto de Souza, Cláudio Lucas Miranda, Andrezza Belluomini Castro, Patrícia Carvalho Garcia-Bonichini

**Affiliations:** Hemocentro do Hospital das Clínicas da Faculdade de Medicina de Botucatu (HCFMB-UNESP), Botucatu, SP, Brazil

**Keywords:** Blood transfusion, Patient blood management, Cardiac surgery, Intraoperative bleeding, Diabetes mellitus

## Abstract

**Background:**

Blood is a biological, irreplaceable, and perishable resource, provided through voluntary, altruistic, and free donation in Brazil. Although blood components are widely used in hospital settings, several challenges persist, including the limited availability of these resources, the high costs associated with their procurement, storage, and transfusion, as well as the risks inherent in the allogeneic transfusion process due to potential transfusion reactions. Therefore, there is a need to focus on Patient Blood Management (PBM) within the Brazilian transfusion system to reduce the need for transfusions, particularly of packed red blood cells, during elective cardiac surgeries.

**Objective:**

To evaluate the risk factors associated with the use of packed red blood cells in elective cardiac surgeries performed at the Hospital das Clínicas, Faculty of Medicine, Botucatu.

**Methods:**

This retrospective study involving 741 individuals was conducted between 2018 and 2021 with the approval of an ethics research committee. Data were analyzed using descriptive statistics, the Chi-square test, and stepwise logistic regression, with the level of significance set at 5 %.

**Results and conclusion:**

Preoperative factors such as female sex (Odds ratio: 9.074; p-value <0.0001), low hematocrit levels (Odds ratio: 7.498; p-value = 0.0034), and the presence of diabetes mellitus (Odds ratio: 1.779; p-value = 0.0318), as well as intraoperative factors such as extracorporeal circulation time greater than 90 min (Odds ratio: 1.68; p-value = 0.0442), were identified as risk factors for excessive bleeding and the need for packed red blood cells during surgery.

## Introduction

Knowing that transfusion is possible exclusively due to blood donation [1], the rational use of this resource must be evaluated and implemented in blood therapy centers. While transfusions are an undeniable need, several challenges arise. The limited availability of blood components, combined with high costs of procurement, storage, and transfusion, as well as the risks associated with the allogeneic transfusion process – such as transfusion reactions [2, 3, 4] - contrasts sharply with the high demand for blood products. This demand is further exacerbated by the constant use of blood components as therapeutic resources, population aging, increasingly restrictive screening criteria [5,6], difficulties in recruiting new donors [3], and festive, climatic and epidemiological events, such as COVID-19 pandemic [7].

Moreover, the irrational use of blood and the failure to minimize unnecessary exposure to blood products [2,8,9] which directly affects already declining stocks, continues to be problems. This issue seems to stem from clinical practices where professionals recommend and approve blood transfusion when other interventions, mainly during the perioperative period, have been shown to be more effective and safer in the treatment of patients [8].

While transfusion of packed red blood cells can be life-saving, it also presents risks to the patient and entails significant costs [2]. These factors disrupt the cost-benefit balance of this type of therapy, underscoring the need for safer and more effective alternatives. In this context, Patient Blood Management (PBM) is particularly relevant given the realities of blood transfusions in Brazil and as recommended by the World Health Organization (WHO) [2].

This program consists of an interdisciplinary, individualized, and multimodal approach based on evidence and medical interventions, when necessary and timely, to maintain the blood mass, minimize the need for allogeneic transfusions, and promote safe and rational use of available blood therapy resources [5,8,10].

To this end, different studies have identified three main strategies or pillars for implementing PBM in healthcare settings: (1) management of the patient's anemia, which includes addressing preoperative anemia through the administration of intravenous or oral iron, for example; (2) minimization of iatrogenic or unnecessary blood loss through blood-sparing surgical techniques, perioperative autologous blood collection and re-transfusion, and the use of hemostatic agents such as antifibrinolytics; and (3) optimization of the patient's specific physiological tolerance to anemia by adhering to transfusion triggers [5,8,9,11].

Regarding the first pillar, literature has shown that the majority of blood transfusions (around 94 %) are due to low preoperative hemoglobin levels (diagnosed in between 20 and 60 % of cases), excessive blood loss during surgery, and iatrogenic transfusion practices [9,12]. These findings are consistent with other studies, which report a prevalence of 22–30 % of preoperative anemia in patients undergoing cardiac surgery. Anemic patients experience worst postoperative outcomes compared to non-anemic patients, including longer hospital stays, higher risk of infectious complications, increased morbidity, and even mortality [6,12].

One the other hand, the implementation of PBM has been associated with improved perioperative outcomes across various types of surgery, including significant reductions in complications and mortality, particularly among cardiac and orthopedic surgery patients [5,13]. In fact, PBM has contributed to enhanced patient safety, cost savings, and a reduction in hospital readmissions [10].

Recent and important studies emphasize the need for perioperative interventions in cardiac surgeries, as these practices substantially reduce transfusion requirements and are associated with better patient health outcomes [8]. Scientific evidence indicates that the need for transfusions increases the risk of morbidity and mortality in these surgeries. As a result, guidelines from the Society of Cardiovascular Anesthesia recommend promoting interventions that increase red blood cell volume to reduce the risk of transfusions of packed red blood cells [13].

In addition to the preexistence of anemia, several other factors may contribute to an increased risk of excessive bleeding as observed in both literature and unpubliched data [14]. These factors include gender 15, 16, age 15, 16, 17, systemic arterial hypertension, diabetes mellitus, platelet count, prothrombin activation time, activated partial thromboplastin activation time [17] and the use of medications such as anticoagulants, antiplatelet agents, and non-steroidal anti-inflammatory drugs. The potential suspension of these medications should be carefully considered for each surgery candidate due to individual predisposition to perioperative bleeding 17, 18.

Similarly, intraoperative factors – those present during the surgical procedure - can also pose a risk for the transfusion of packed red blood cells. For example, the use of extracorporeal circulation and its duration are already known to be high-risk factors for bleeding, directly influencing the number of allogeneic blood units transfused 16, 17, 19. Among the various intraoperative measures identified in the literature, the use of topical or systemic agents such as tranexamic-acid and epsilon-aminocaproic acid stands out. When used appropriately, these agents help prevent excessive blood loss during cardiac surgery 5, 20, 21.

Therefore, this study aimed to evaluate the risk factors associated with the use of packed red blood cells in elective cardiac surgeries from a PBM perspective. It sought to evaluate the preoperative and intraoperative variables related to increased bleeding during these procedures, considering their significant implications for in-hospital practices and medical protocols in relation to patient care and the use of blood components.

## Methods

All activities carried out were approved by the Hospital das Clínicas da Faculdade de Medicina de Botucatu (HCFMB) Research Ethics Committee (opinion 5627,357) recognized by the National Research Ethics Commission, and in accordance with the guidelines outlined in Resolution 466/12 of the National Health Council. This study was conducted at the Transfusion Agency of the Blood Center at the HCFMB.

This retrospective study is based on data collected from surgical and electronic medical records focusing on preoperative and intraoperative factors associated with the use of packed red blood cells in elective cardiac surgeries performed between 2018 and 2021 (Table 1). It is important to note that the reference values used for statistical analysis are those adopted by the hospital.

**Table 1:** Preoperative and intraoperative variables and criteria associated with excessive bleeding risk.

Variables	Operational definitions and criteria for excessive bleeding risk as described in the literature and reference values
Age	Obtained through registration in electronic medical records in preliminary evaluation to surgery. Bleeding risk: elderly^15^, ^16^, ^17^
Sex	Obtained through registration in electronic medical records. Bleeding risk: male^15^, ^16^ and female
Systemic arterial hypertension (SAH)	Verified as a clinical diagnosis prior to surgery and previous pathological history recorded in the electronic medical records. Bleeding risk: occurrence^17^
Diabetes Mellitus (DM)	Verified as a clinical diagnosis prior to surgery and previous pathological history recorded in the electronic medical record. Bleeding risk: not characterized^17^
Hemoglobin (Hb) Levels	Obtained from the last laboratory test collected prior to surgery (up to 10 days) and recorded in the electronic medical record. Risk of bleeding: anemia present^6^. Reference values: Birth (18.0 ± 4.0 g/dL), 3 days (18.0 ± 3.0 g/dL), 1 month (14.0 ± 2.5 g/dL), 2 months (11.2 ± 1.8 g/dL), 3 to 6 months (12.6 ± 1.5 g/dL), 1 year (12.6 ± 1.5 g/dL), 2 to 6 years (12.6 ± 1.5 g/dL), 6 to 12 years (12.5 ± 1.5 g/dL)^22^ adult women (14.0 ± 2.0) and adult men (15.5 ± 2.0 g/dL). All adopted by HCFMB
Hematocrit (Ht) Levels	Obtained from the last laboratory test collected prior to surgery (up to 10 days) and recorded in the electronic medical record. Bleeding risk: below 30 %^15^, ^17^. Reference values: Birth (60 ± 15 %), 3 days (56 ± 11 %), 1 month (43 ± 10 %), 2 months (35 ± 7 %), 3 to 6 months (35 ± 5 %), 1 year (34 ± 4 %), 2 to 6 years (37 ± 3 %), 6 to 12 years (40 ± 5 %), adult women (41 ± 5 %)^22^ and adult men (47 ± 6 %). All adopted by HCFMB
Platelet Count (PLT)	Obtained from the last laboratory test collected prior to surgery (up to 10 days) and recorded in the electronic medical record. Bleeding risk: below 150,000 cells/mm³^17^. Reference value adopted by HCFMB: 140,000 to 440,000 10³/mm³
Prothrombin activation time	Obtained from the last laboratory test collected prior to surgery (up to 10 days) and recorded in the electronic medical record. Bleeding risk: above 14 s^17^. Reference value adopted by HCFMB: above 14 s, approximately International Normalized Ratio > 1.2
Activated partial thromboplastin time (APTT)	Obtained from the last laboratory test collected prior to surgery (up to 10 days) and recorded in the electronic medical record. Bleeding risk: above 40 s^17^. Reference value adopted by HCFMB: above 40 s, with a normal ratio of ≥ 0.70 to 1.25; above > 1.25
Antiplatelet therapy	Verified in medication prescriptions prior to surgery (up to 5 days) recorded in the electronic medical record. Examples of medications: non-steroidal anti-inflammatory drugs (NSAIDs), such as acetylsalicylic acid (AAS), dipyrone and paracetamol; blood thinners, such as warfarin, heparin, and enoxaparin; and antiplatelet agents, such as ketoprofen, clopidogrel and cilostazol. Bleeding risk: non-interruption or interruption for <5 days before surgery^11^, ^16^, ^17^, ^18^
History of Covid-19	Obtained in the past history in the years 2020 and 2021 and prior to surgery. Registered in electronic medical records
Type of surgery performed	Recorded in surgical map and electronic medical record. The surgeries analyzed were: Communication closure (intraventricular or interatrial); pacemaker implantation; valve prosthesis implantation; and myocardial revascularization. Risk of bleeding: when ECC is used^16^, ^17^
Classification as reoperation	Record in surgical map and history prior to surgery recorded in electronic medical records. Risk of bleeding: when present^16^
Need for Extracorporeal Circulation	Registered in electronic medical record. Risk of bleeding: when ECC is used^16^, ^17^
Extracorporeal Circulation Time	Obtained through electronic medical records after surgery. Bleeding risk: prolonged ECC, around 90–110 min^15^. Reference Value used by HCFMB: normal up to 90 min^19^
Activated Clotting Time (ACT)	Obtained through electronic medical records after surgery. Reference value used by HCFMB: normal between 80–120 s^19^
Therapy with systemic hemostatic agents	Recorded in electronic medical records with prescription from 5 days before surgery until 1 day after surgery. Medications: tranexamic-acid, epsilon-aminocaproic acid^4^, ^5^, ^18^, ^20^

*Source:* Adapted from Braga & Brandão, 2018, and Souza, 2022 (unpublished data).

The main exclusion criteria were unsatisfactory or incomplete data, meaning patients who did not have all the necessary information for the required parameters; patients with test results that were older than ten days prior to surgery, based on the clinical validity determined by the Laboratory of Clinical Analysis and the Blood Center of the HCFMB, patients who underwent two elective cardiac surgeries simultaneously; and patients who died during surgery, as this event negatively impacted the complete documentation of surgical information and its relevance to the study.

The collected data were subjected to a descriptive analysis, which included the calculation of mean, standard deviation, minimum, maximum, and median values for quantitative variables, as well as frequencies and percentages for categorized variables. The chi-square test was used to assess associations between the use or non-use of packed red blood cells and the other study variables.

Subsequently, a Logistic Regression (stepwise analysis) was conducted, with the use or non-use of packed red blood cells as the dependent variable and the other variables as explanatory, in order to identify risk factors for the event. A 5 % significance level was applied to all tests, using the SAS 9.4 computer software.

## Results

The present study analyzed data from all patients undergoing elective cardiac surgery and who met the inclusion criteria, with a total number of 741 patient records reviewed. The patient’s ages ranged from 1–95 years, with a mean age of 61.79 years, a standard deviation of 15.19, and a median age of 63.00 years. These results indicate a higher prevalence of advanced age. Regarding intraoperative parameters, myocardial revascularization surgery was the most common, accounting for 50.88 % of all procedures.

Additionally, more than half of the surgeries (53.31 %) involved the use of extracorporeal circulation, a known major risk factor for bleeding, as reported in the literature. Descriptive statistics for the remaining parameters are presented in Table 2. It is important to note that all patients met the inclusion criteria, and their reference values were based on the hospital's practice for surgeries.

**Table 2:** Descriptive values of preoperative and intraoperative variables and their association with the use or not of packed red blood cells during elective cardiac surgery.

Variable	Subvariable	n (%)	Did not receive packed red blood cells	Received packed red blood cells	p-value
Sex	Female Male	266 (35.9 %) 475 (64.1 %)	149 (29.3 %) 360 (70.7 %)	117 (50.4 %) 115 (49.6 %)	<0.0001⁎⁎
Systemic arterial hypertension	Present Absent	479 (64.64 %) 262 (35.36 %)	323 (63.5 %) 186 (36.5 %)	156 (67.2 %) 76 (32.8 %)	0.3178
Diabetes mellitus	Present Absent	241 (32.52 %) 500 (67.48 %)	153 (30.1 %) 356 (69.9 %)	88 (37.9 %) 144 (62.1 %)	0.0339⁎
Hemoglobin	Below Above Normal	318 (43.03 %) 13 (1.76 %) 408 (55.21 %)	184 (36.3 %) 9 (1.8 %) 314 (61.9 %)	134 (57.8 %) 4 (1.7 %) 94 (40.5 %)	<0.0001⁎⁎
Hematocrit	Below Above Normal	320 (43.36 %) 14 (1.90 %) 404 (54.74 %)	188 (37.2 %) 6 (1.2 %) 312 (61.7 %)	132 (56.9 %) 8 (3.4 %) 92 (39.7 %)	<0.0001⁎⁎
Platelets	Below Above Normal	39 (5.28 %) 8 (1.08 %) 691 (93.63 %)	30 (5.9 %) 4 (0.80 %) 472 (93.3 %)	9 (3.9 %) 4 (1.7 %) 219 (94.4 %)	0.2774
Prothrombin activation time	Above Normal	66 (8.91 %) 675 (91.09 %)	49 (9.6 %) 460 (90.4 %)	17 (7.3 %) 215 (92.7)	0.3082
Activated partial thromboplastin time	Below Above Normal	246 (33.20 %) 8 (1.08 %) 487 (65.72 %)	176 (34.6 %) 7 (1.4 %) 326 (64.0 %)	70 (30.2 %) 1 (0.4 %) 161 (69.4 %)	0.2298
Non-steroidal anti-inflammatory drugs - total	Present Absent	397 (53.58 %) 344 (46.42 %)	254 (49.9 %) 255 (50.1 %)	143 (61.6 %) 89 (38.4 %)	0.003⁎
Non-steroidal anti-inflammatory drugs - acetylsalicylic acid	Present Absent	352 (47.50 %) 389 (52.50 %)	224 (44.0 %) 285 (56.0 %)	128 (55.2 %) 104 (44.8 %)	0.0048⁎
Non-steroidal anti-inflammatory drugs - dipyrone	Present Absent	51 (6.88 %) 690 (93.12 %)	35 (6.9 %) 474 (93.1 %)	16 (6.9 %) 216 (93.1 %)	0.9919
Non-steroidal anti-inflammatory drugs - paracetamol	Present Absent	22 (2.97 %) 719 (97.03 %)	16 (3.1 %) 493 (96.9 %)	6 (2.6 %) 226 (97.4 %)	0.6786
Anticoagulant - total	Present Absent	68 (9.18 %) 673 (90.82 %)	49 (9.6 %) 460 (90.4 %)	19 (8.2 %) 213 (91.8 %)	0.5298
Anticoagulants - Warfarin	Present Absent	3 (0.40 %) 738 (99.60 %)	1 (0.2 %) 508 (99.8 %)	2 (0.9 %) 230 (99.1 %)	0.1858
Anticoagulants - enoxaparin	Present Absent	58 (7.83 %) 683 (92.17 %)	44 (8.6 %) 465 (91.4 %)	14 (6.0 %) 218 (94.0 %)	0.22
Anticoagulants - heparin	Present Absent	8 (1.08 %) 733 (98.92 %)	5 (1.0 %) 504 (99.0 %)	3 (1.3 %) 229 (98.7 %)	0.7042
Antiaggregant - total	Present Absent	14 (1.89 %) 727 (98.11 %)	9 (1.8 %) 500 (98.2 %)	5 (2.2 %) 227 (97.8 %)	0.7197
Antiplatelet agent - ketoprofen	Present Absent	1 (0.13 %) 740 (98.87 %)	1 (0.20 %) 508 (99.8 %)	0 232 (100 %)	0.4993
Antiplatelet agent - Cilostazol	Present Absent	3 (0.40 %) 738 (99.60 %)	3 (0.6 %) 506 (99.4 %)	0 232 (100 %)	0.2413
Antiplatelet agent - clopidogrel	Present Absent	12 (1.62 %) 729 (98.38 %)	7 (1.4 %) 502 (98.6 %)	5 (2.2 %) 227 (97.8 %)	0.4354
COVID-19	Present Absent	7 (0.94 %) 734 (99.06 %)	7 (1.4 %) 502 (98.6 %)	0 232 (100 %)	0.0727
Type of surgery	Communication closure Pacemaker implant Valve prosthesis implantation Revascularization of the myocardium	30 (4.05 %) 229 (30.9 %) 105 (14.17 %) 377 (50.88 %)	12 (2.4 %) 228 (44.8 %) 40 (7.9 %) 229 (45.0 %)	18 (7.8 %) 1 (0.4 %) 65 (28.0 %) 148 (63.8 %)	<0.0001⁎
Reoperation	Present Absent	115 (15.52 %) 626 (84.48 %)	101 (19.8 %) 408 (80.2 %)	14 (6.0 %) 218 (94.0 %)	<0.0001⁎
Extracorporeal circulation	Present Absent	395 (53.31 %) 346 (46.69 %)	191 (37.5 %) 318 (62.5 %)	204 (87.9 %) 28 (12.1 %)	<0.0001⁎
Extracorporeal circulation time	Above Normal	245 (63.97 %) 138 (36.03 %)	108 (58.1 %) 78 (41.9 %)	137 (69.5 %) 60 (30.5 %)	0.0194
Activated clotting time	Below Above Normal	5 (1.29 %) 249 (64.01 %) 135 (34.70 %)	2 (1.0 %) 118 (61.8 %) 71 (37.2 %)	3 (1.5 %) 131 (66.2 %) 64 (32.3 %)	0.5723
Systemic hemostatic agents - total	Present Absent	2 (0.27 %) 739 (99.73 %)	0 509 (100 %)	2 (0.9 %) 230 (99.1 %)	0.0359⁎
Systemic hemostatic agents - Tranexamic acid	Present Absent	1 (0.13 %) 740 (99.87 %)	0 509 (100 %)	1 (0.4 %) 231 (99.6 %)	0.1383
Systemic hemostatic agents - Epsilon-aminocaproic acid	Present Absent	1 (0.13 %) 740 (99.87 %)	0 509 (100 %)	1 (0.4 %) 231 (99.6 %)	0.1383

The categories ‘below’, ‘above’ and ‘normal’ are related to reference values as adopted by the HCFMB.

⁎ Statistically significant values, *p*-value <0,05.

⁎⁎ Statistically significant values, *p*-value <0,0001.

By analyzing the parameters studied and the number of blood bags used in elective cardiac surgeries, an association was found between these factors during the observed period, as shown in Table 2. It is important to note that, only for this statistical analysis, blood bags used to fill the heart-lung system during extracorporeal circulation (when applicable) were excluded from the total count. This was done to ensure that the results solely reflect the transfusion of red blood cell concentrates required to meet the patient's needs in the event of transfusion during the surgical procedure.

Logistic regression was performed using stepwise analysis, with the odds ratio (OR) estimated based on the use or not of packed red blood cells as the response variable and the other factors as explanatory variables. The goals were to identify risk factors for the event. Only four of the evaluated parameters were classified as risk factors, despite other factors being identified as significant by the chi-square test.

The factors associated with a higher risk of requiring transfusions during elective cardiac surgery included female sex (OR: 9.074; 95 % Confidence interval [95 % CI]: 5.218; p-value <0.0001), low hematocrit (OR 7.498; 95 %CI 4.362; p-value = 0.0034), presence of diabetes mellitus (OR 1.779; 95 % CI 1.052; p-value = 0.0318), and cardiopulmonary bypass time exceeding 90 min (OR 1.68; 95 % CI 1.013; p-value = 0.0442).

## Discussion

As the prescription for packed red blood cells has become more prevalent in recent years [5,6], the need for a more attentive and careful approach is urgent. This is not only due to the significant financial burden it places on the hospital system and, in many cases, on the government, but also because of the progressive decline in blood donations [3].

Therefore, there is an urgent need to evaluate the factors associated with the use of packed red blood cells during elective cardiac surgeries, in order to identify risk of excessive intraoperative bleeding and the need for transfusions related to these variables. This highlights the importance of understanding PBM Programs [4, 5, 6,9], which are crucial for promoting a significant reduction in transfusions. Such programs can help to avoid unnecessary costs, mitigate risk to patients undergoing transfusion therapy, and ensure better control of blood stocks in the blood bank. It is also important to note the data obtained in this study may aid medical practice by providing insights for surgery teams to more carefully consider patients who present one or more of the risk factors for needing transfusions.

The current research yielded preliminary results indicating a significant relationship between the use or not of packed red blood cells and the following parameters: sex, diabetes mellitus, hemoglobin, hematocrit, non-steroidal anti-inflammatory drugs (acetylsalicylic-acid), type of surgery, reoperation, extracorporeal circulation, duration of extracorporeal circulation, and the use of systemic hemostatic agents (Table 2).

Although all these parameters showed some correlation with the use or not of packed red blood cells during the surgical procedure, a secondary logistic regression analysis revealed that only four were associated with significant risk related to the studied outcome. These variables were female sex, diabetes mellitus, hematocrit below the reference value for age adopted by the study hospital (Table 2) and extracorporeal circulation time exceeding 90 min. This factor may have arisen due to the small sample sizes of certain variables (presence or absence, above, normal or below) which were too limited to establish a reliable correlation when compared to the total number of participants in the study.

Nevertheless, although the current study showed a male predominance (64.1 %) of advanced age patients, with a median age of 63 years, which aligns with similar studies 15, 17 that identify these as risk factors for excessive perioperative bleeding, an OR 9.074 (p-value <0.0001) was observed for females. While many studies do not support this finding, it may be closely linked to the higher prevalence of anemia in women [23], which could be associated with sex-specific factors such as menstruation, pregnancy, and lactation.

Despite this, the present study observed an OR 7.498 (p-value = 0.0034) for hematocrit levels below the reference values adopted by the study hospital, strongly suggesting the presence of preoperative anemia. This finding is further supported by the significant data observed for hemoglobin (p-value = 0.0001) and by the comparison of the number of individuals with hemoglobin and hematocrit levels below the reference values who received packed red blood cells during surgery versus those who did not. Specifically, 134 (57.8 %) and 132 (56.9 %) individuals with low hemoglobin and low hematocrit, respectively received blood transfusions, compared to 184 (36.3 %) and 188 (37.2 %) who did not.

This situation is further supported by discussions surrounding PBM Programs, which focus on treating anemia, particularly iron deficiency, given that anemia is widely recognized as an important factor contributing to the need for transfusions 5, 6, 11, 15, 17. An additional key factor in this context is the association between preoperative anemia and comorbidities, advanced age, and female gender - all of which are identified as risk factors for adverse outcomes following cardiac surgery [24].

Regarding the duration of extracorporeal circulation exceeding the reference values adopted by the hospital (over 90 min), with an OR 1.68 (p-value = 0.0442), a relationship is observed with findings from other studies 15, 16. This is consistent with the preliminary results of the present study, which also showed significant associations with the use of extracorporeal circulation (p-value <0.001) and the type of surgery performed (p-value <0.001). These findings may be particularly influenced by myocardial revascularization surgery followed by valve prosthesis implantation, which are associated with a prolonged use of extracorporeal circulation.

Cardiopulmonary bypass is potentially one of the main causes of bleeding during the perioperative period due to coagulation disorders and the activation of fibrinolysis, factors that are further exacerbated by prolonged usage. This is also considered one of the primary contributors to bleeding in cardiac surgeries involving extracorporeal circulation 14, 21. Additionally, it is known that patients with a hematocrit below 40 % who require extracorporeal circulation have a higher likelihood of needing blood transfusions during myocardial revascularization surgery, which is a concern regarding the use of blood components [4].

Finally, although the presence of diabetes mellitus was classified for the present study with OR 1.779 (p-value = 0.0318), a finding that contrasts with what is generally reported in the literature [17], this comorbidity is considered prevalent in complicated patients undergoing cardiac surgery [25]. Furthermore, its prevalence is significantly associated with the group of patients who receive transfusions, as seen in various studies, whether related to other diseases or not 26, 27.

Thus, it is evident that hyperglycemia is closely linked to cardiovascular events due to chronic inflammation [28], as well as to the occurrence of systemic events, particularly those related to atherosclerosis, which results from the severity and duration of insulin resistance (IR) in diabetic patients 29, 30. Pre-existing anemia 27, 31 and altered hemostatic events further contribute to this relationship.

Although the literature presents an extensive range of issues regarding diabetes mellitus, it is still not possible to prove the relationship between the presence of this comorbidity and the need for transfusions, thus raising speculations on the subject as presented below:

(1) Anemia: The first proposal highlights the need for transfusions due to pre-existing anemia in diabetic patients. Type 2 diabetes mellitus, due to the hemodilution caused by high glucose levels in the body [31], contributes to reduced hemoglobin and hematocrit values, indicating anemia 27, 31. Two additional sub-proposals included in this item focus on the role of erythropoietin, which may be insufficient in the presence of this comorbidity, especially when considering the hypoxia induced by the disease [31] and the reduction in hemoglobin levels. Furthermore, diabetic patients are more likely to have iron deficiency due to impaired intestinal absorption, which further contributes to anemia, along with an impaired ability to produce erythropoietin in response to decreased hemoglobin [27].
(2) Vascular complications: It is well established that insulin resistance is strongly associated with endothelial dysfunction, especially when proinsulin levels are elevated, which stimulates the production of plasminogen activator inhibitors and impairs fibrinolysis 29, 30. A study conducted on patients undergoing transcatheter aortic valve implantation found that clinically significant bleeding complications were linked to the presence of diabetes mellitus and associated with vascular diameters [32]. Additionally, the prevalence of hyperfibrinogenemia in patients with type 2 diabetes mellitus, where fibrinogen levels contribute to an increasing cardiovascular risk [33], was observed. This may indirectly relate to the need for transfusions, although this connection has not been extensively discussed in the literature.
(3) Women: Carotid atherosclerosis caused by diabetes mellitus was found to be more prevalent in women than in men, along with increased oxidative stress and endothelin-1 levels, which contribute to vasoconstriction and platelet aggregation. This leads to a more pro-thrombotic fibrin profile, promoting the formation of denser fibrin clots and prolonged fibrinolysis [34], as well as a higher risk of vascular complications, which are more pronounced in women [35]. These factors are associated with the gender-related findings on the use of packed red blood cells in the present study. Therefore, an important aspect to highlight about women is their concurrent incidence of diabetes mellitus. The explanation for this finding lies in the fact that diabetes mellitus diminishes the development of heart disease and nephropathies, predisposing them to higher cardiovascular risks compared to men. This is related to genetic factors, endocrine disorders, psychosocial stress, and differing sociocultural behavior between women and men [34].

Although all of the factors mentioned above are clinically significant, it is important to note that, despite the patients in the present study being under medical care shortly before surgery, given that the procedures were classified as elective, many of the individuals were likely not admitted to the hospital immediately prior to the procedure. This may have led to a lack of control over their daily care, particularly concerning the proper use of medication and adherence to the recommended diet for diabetes mellitus.

In addition, body mass index calculations, blood glucose measurements before surgery, and classification between type 1 and 2 diabetes mellitus were not provided, highlighting the absence of important data to better understand the relationship between diabetes mellitus and the need for transfusions.

Finally, it is important to emphasize that, although the literature describes the connection between diabetes mellitus and complications in cardiovascular diseases, there are still no pathophysiological explanations that establish a causal link between the presence of this comorbidity and the need of transfusions, even though this relationship has been observed. Therefore, experimental studies are necessary to offer a clearer explanation of the data observed in the present study.

## Conclusion

Preoperative risk factors, such as female sex, decreased hematocrit, and presence of diabetes mellitus, along with intraoperative risk factors, such as the use of extracorporeal circulation for >90 min, have been shown to be associated with the use of packed red blood cells in elective cardiac surgeries conducted between 2018 and 2021.

## Financing

This research did not receive any specific grants from public, commercial, or non-profit sector funding agencies.

## Conflicts of interest

During the preparation of this work, the author(s) used ChatGPT to improve the translation into English. After using this tool/service, the author(s) reviewed and edited the content as needed and take(s) full responsibility for the content of the publication.
